# 

**Published:** 1867-04

**Authors:** 


### CONTENTS.

### ORIGINAL COMMUNICATIONS.

PAGE.

ARTICLE I.—An Address delivered at the last meeting of the Medical Association of Georgia. By the late President, Dr. J. T. Banks, on retiring from the Chair....... 49

ARTICLE II.—Pathology and treatment of Milk-sickness. By W. B. Miller, M. D., of Calhoun, Ky.......... 57

ARTICLE III.—Trismus Nascentium. By Jno. M. Langhorn, M. D., of Uniontown, Ala.......... 61

ARTICLE IV.—Extracts from the minutes of the Atlanta Medical society. By Dr. W. S. Armstrong, Demonstrator of Anatomy, Atlanta Medical College............ 63

### SELECTIONS.

Case of Vicarious Menstruation.......... 67

On the management of Weak New-born Infants.......... 71

Is a Crying Babe necessarily Colicky.......... 73

Suture of the Flap, after extract of Cataract.......... 77

Bloodletting then and now.......... 79

### EDITORIAL AND MISCELLANEOUS.

The Atlanta Medical College—Its Prospects.......... 85

The Medical Association of Georgia.......... 87

Hypodermic Administration of Medical Agents.......... 87

American Medical Association.......... 92

Bibliographical.......... 94

Meteorological Table.......... 96

